# Neuroimaging biomarkers of small vessel disease in cerebral amyloid angiopathy‐related intracerebral hemorrhage

**DOI:** 10.1111/cns.14098

**Published:** 2023-02-05

**Authors:** Mengke Zhang, Ruiwen Che, Wenbo Zhao, Hailiang Sun, Changhong Ren, Jin Ma, Wenbo Hu, Milan Jia, Chuanjie Wu, Xin Liu, Xunming Ji

**Affiliations:** ^1^ Department of Neurology, Xuan Wu Hospital Capital Medical University Beijing China; ^2^ Department of Neurology, Beijing Shijitan hospital Capital Medical University Beijing China; ^3^ Beijing Key Laboratory of Hypoxia Conditioning Translational Medicine, Xuan Wu Hospital Capital Medical University Beijing China; ^4^ Department of Neurosurgery Beijing Fengtai You'anmen Hospital Beijing China; ^5^ Department of Radiology, Beijing Chaoyang Hospital Capital Medical University Beijing China; ^6^ Department of Neurosurgery, Xuan Wu Hospital Capital Medical University Beijing China

**Keywords:** cerebral amyloid angiopathy, cerebral hemorrhage, cerebral small vessel diseases, neuroimaging

## Abstract

**Aims:**

The significance of the correlation of computed tomography (CT)–based cerebral small vessel disease (SVD) markers with the clinical outcomes in patients with cerebral amyloid angiopathy (CAA)‐related intracerebral hemorrhage (ICH) remains uncertain. Thus, this study aimed to explore the relationship between SVD markers and short‐term outcomes of CAA‐ICH.

**Methods:**

A total of 183 patients with CAA‐ICH admitted to the Xuanwu Hospital, and Beijing Fengtai You'anmen Hospital, from 2014 to 2021 were included. The multivariate logistic regression analysis was performed to identify the correlation between SVD markers based on CT and clinical outcomes at 7‐day and 90‐day.

**Results:**

Of the 183 included patients, 66 (36%) were identified with severe SVD burden. The multivariate analysis showed that the total SVD burden, white matter lesion (WML) grade, and brain atrophy indicator were independent risk factors for unfavorable outcomes at 90‐day. The brain atrophy indicator was independently associated with mortality at 90‐day. Severe cortical atrophy was significantly associated with early neurological deterioration.

**Conclusions:**

The neuroimaging profiles of SVD based on CT in patients with CAA‐ICH might predict the short‐term outcome more effectively. Further studies are required to validate these findings and identify modifiable factors for preventing CAA‐ICH development.

## Graphical abstract

The significance of the correlation of computed tomography–based cerebral small vessel disease (SVD) markers with the clinical outcomes in patients with cerebral amyloid angiopathy (CAA)‐related intracerebral hemorrhage (ICH) remains uncertain. In our study, of the 183 included patients [with average age, 71 years; and male participants, 98 (54%)], 66 (36%) were identified with severe SVD burden. The multivariate analysis showed that the total SVD burden (*p* < 0.001, OR, 2.74; 95% CI, 1.61–4.67), white matter lesion (WML) grade (*p* < 0.001, OR, 8.93; 95% CI, 2.63–30.38), and brain atrophy indicator (*p* = 0.009, OR, 0.76; 95% CI, 0.61–0.93) were independent risk factors for unfavorable outcomes at 90‐day. The brain atrophy indicator (*p* = 0.027, OR, 0.78; 95% CI, 0.63–0.97) was independently associated with mortality at 90‐day. Severe cortical atrophy (*p* = 0.031, OR, 2.98; 95% CI, 1.10–8.04) was significantly associated with early neurological deterioration within 7 days. Thus, considering cerebral SVD on baseline CT when determining prognosis in patients in the acute phase with CAA‐ICH is promising.

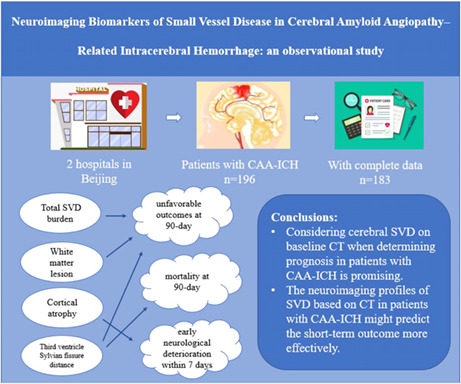

## 1 INTRODUCTION

Cerebral amyloid angiopathy (CAA) is a cerebral small‐vessel disease caused by the deposition of β‐amyloid in the walls of arteries, arterioles, and capillaries in the cerebral cortex and overlying leptomeninges.^1^ CAA is well recognized as a common cause of spontaneous lobar intracerebral hemorrhage (ICH), which is the most devastating form of stroke, with a considerable death rate approaching 50%.^2^ Although patients with CAA‐ICH have mild symptoms, a higher risk of recurrent and/or multiple hemorrhages may result in higher morbidity and mortality.^3^ Unfortunately, no effective treatment and prevention methods are available. The surgical treatment during the acute phase is controversial.^4^ Therefore, understanding the outcomes of the disease and the prognostic factors may help make clinical decisions.

Previous studies demonstrated a stronger correlation between ICH and underlying SVD. The neuroimaging abnormalities assessed using magnetic resonance imaging (MRI)/CT scan can lead to an unfavorable functional outcome and ICH recurrence.^5^, ^6^, ^7^, ^8^, ^9^, ^10^, ^11^ The characteristic neuroimaging biomarkers of SVD are also observed in CAA, such as cerebral microbleeds, cortical superficial siderosis, white matter lesion (WML), enlarged perivascular space, lacunes, and cerebral atrophy.^12^ Whether the underlying SVD burden is a significant predictor of unfavorable short‐term outcome in patients with CAA‐ICH is not certain to date. Compared with emergency MRI, CT brain scans are still the main approach for patients with ICH in the acute phase in most hospitals, especially in primary hospitals. Furthermore, the results of MRI in the subacute phase may be influenced due to the enlarged edema and hematoma. Hence, exploring the significance of CT markers for CAA‐ICH in the hyperacute and acute phases is essential.

Hence, this study was performed to investigate whether the preexisting SVD features on baseline CT had a prognostic significance in patients with CAA‐ICH to improve their applicability in clinical practice.

## 2 METHODS

### 2.1 Study design and patient selection

This study retrospectively reviewed patients with spontaneous lobar hemorrhage in the Department of Neurology or Neurosurgery, Xuanwu Hospital, Capital Medical University, and Beijing Fengtai You'anmen Hospital, from 2014 to 2021. The design of this study was approved by the research ethics committee in both hospitals for collecting information. Informed consent was obtained from patients or legal representatives. The data were collected in a double‐blind manner.

The inclusion criteria were as follows: (1) diagnosis of ICH confirmed by brain CT scan within 72 h of ictus; (2) patients diagnosed with CAA‐ICH, according to the modified Boston criteria (Version 1.5)^13^; and (3) patients with qualified imaging and follow‐up data. The exclusion criteria were as follows: Patients with (1) preexisting severe neurological deficit [modified Rankin Scale (mRS) score ≥3] and (2) history of tumors and other diseases that influenced the clinical outcome.

Whether the patient underwent surgery or other conservative treatments was based on the Guidelines for the Management of Spontaneous ICH.^14^

### 2.2 Clinical data collection

The medical records of patients were collected, including age, sex, comorbid conditions (history of hypertension, atrial fibrillation, diabetes mellitus, hyperlipidemia, coronary artery disease, prior ischemic stroke or transient ischemic attack, or ICH), toxic habits (smoking or alcohol), use of medications (antiplatelets, anticoagulants, and antihypertensives), systolic and diastolic blood pressure, Glasgow Coma Scale (GCS) score, and National Institutes of Health Stroke Scale (NIHSS) score on admission.

### 2.3 Imaging analysis

All patients underwent noncontrast head CT scans on admission using standardized techniques (recommended slice thickness, 5 mm). All imaging data were assessed by two experienced radiologists blinded to the clinical outcome and other clinical information of patients. They were supposed to achieve a consensus in the case of any disagreement.

On account of irregular hematoma, the focal areas for each layer were multiplied by slice thickness to yield ICH volumes. The WML grade was rated using the Van Swieten Scale (range 0–4). Lacunes were defined as subcortical rounded or ovoid, fluid‐filled (with cerebrospinal fluid signal intensity) lesions of 3–15 mm in diameter, consistent with a previous cerebral infarct or hemorrhage. Severe WMLs were considered if the Van Swieten Scale score was ≥2 in either anterior or posterior periventricular white matter. The cerebral atrophy was recorded on the contralateral side of the hematoma by four parameters. Two linear measurements comprised frontal ratio and third ventricle Sylvian fissure distance, which were measured as described earlier.^7^ A higher frontal ratio and a shorter third ventricle Sylvian fissure distance, separately, proved more advanced cerebral atrophy. We evaluated central and cortical brain atrophy using a 3‐point scale as previously described (none, modest, and severe).^7^ The SVD burden score (0–3) was assessed as previously described: 1 point was assigned for (1) severe WML, (2) severe (≥2) lacunes, and (3) presence of severe deep or cortical brain atrophy.^11^ Severe SVD burden was considered if the score was ≥2, and severe brain atrophy was considered if the scale of visual rating was ≥2 in deep or cortical regions.

### 2.4 Outcome assessment

Four clinical prognostic end points were assessed: early neurological deterioration (END), hematoma expansion (HE) within 7 days, unfavorable functional outcome, and mortality within 3 months. END was defined as an increase of four or more points in NIHSS score or a decrease of two or more points in GCS score within 7 days of enrollment. HE was defined as an absolute increase in hematoma volume of >12.5 ml or a relative increase of >33% within 3 days.^15^ Unfavorable functional outcome was measured at 90‐day with an mRS score of 4–6.

### 2.5 Statistical analysis

All data were analyzed using SPSS, version 25.0. Continuous variables were expressed as mean ± standard deviation (SD) or median and interquartile range (IQR) based on their distribution and results of the Kolmogorov–Smirnov test. They were compared using the *t* test or Mann–Whitney *U* test depending on whether the variables were normally distributed. The categorical variables were presented as percentages and compared using Pearson's chi‐square test or continuous‐correction chi‐square test as appropriate.

We explored the role of each SVD marker individually and the total SVD burden based on brain CT. The univariate logistic regression analysis was performed to assess the relationship between SVD neuroimaging markers and clinical outcomes. To address any concealed confounder, we included confounding variables with a *p* value <0.10 in the multivariate analysis. Previous studies showed that the baseline characteristics and other factors might have an impact on the clinical outcome after ICH.^7^, ^8^, ^11^ A *p* value <0.05 indicated statistically significant differences.

## 3 RESULTS

**FIGURE 1:**
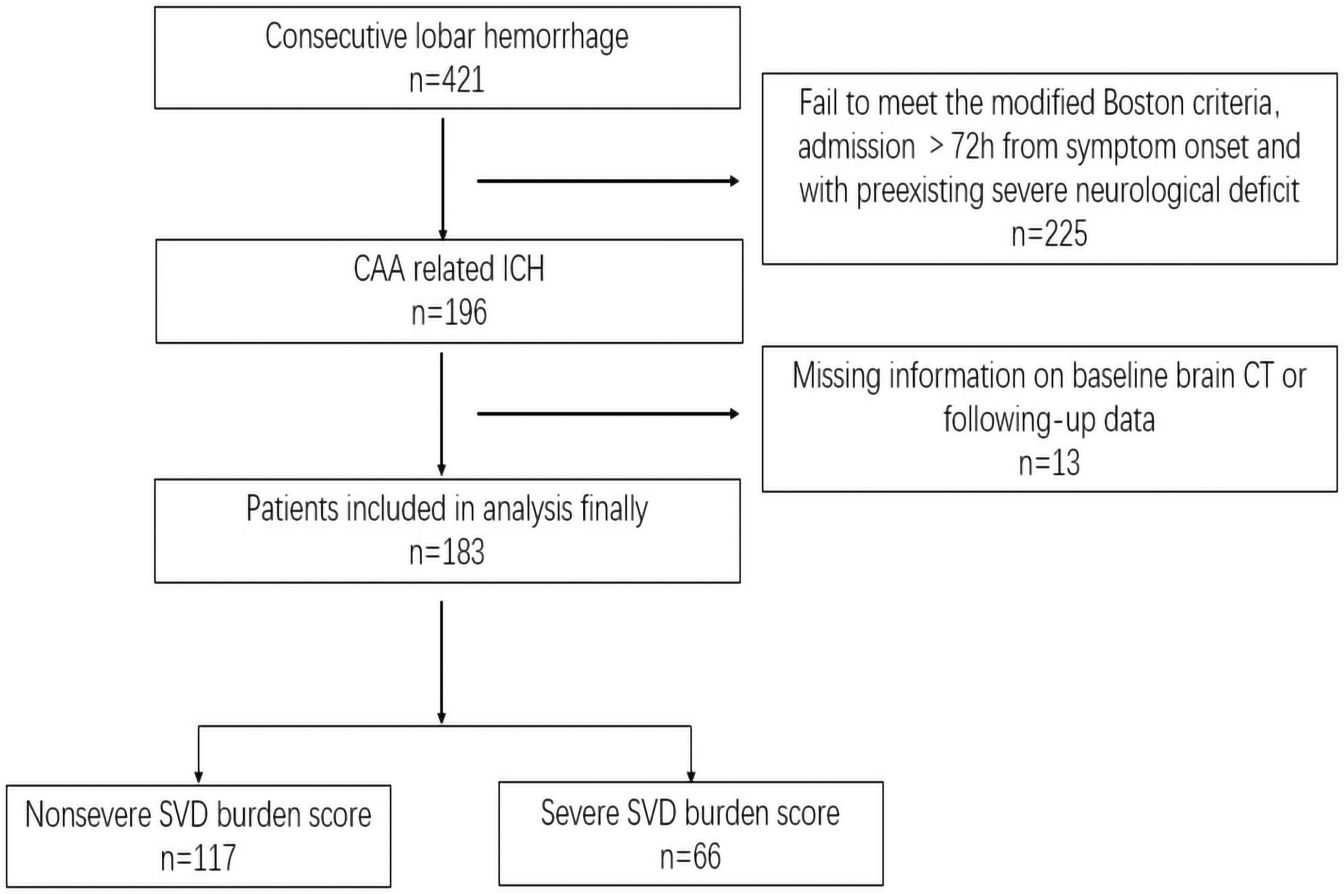
Study flowchart

A total of 196 patients with CAA‐ICH who initially met our criteria were admitted to Xuanwu Hospital, Capital Medical University, and Beijing Fengtai You'anmen Hospital between 2014 and 2021. Of these, 183 patients with complete data (including 2 with CAA with supportive pathology, 66 with probable, and 115 with possible CAA) were finally included. The flowchart of this study is presented in Figure 1.

### 3.1 Patient characteristics

The average age of patients was 71 years, and 98 (54%) participants were male. The median baseline GCS score was 14 (IQR, 11–15), the median NIHSS score was 4 (IQR, 1–15), and the median hematoma volume was 27.02 (IQR, 14.04–51.86) cm^3^. The baseline demographic and clinical characteristics of the included participants are summarized in Table 1.

**TABLE 1:** Demographic and clinical characteristics of participants

Baseline characteristics	Patients included in the analysis *n* = 183	Nonsevere SVD burden score *n* = 117	Severe SVD burden score *n* = 66	*p* value
Demographics				
Age (year)	71 (64–79)	68 (63–76)	77 (70–82)	<0.001
Male	98 (54)	60 (51)	38 (58)	0.412
Medical history				
Hypertension	111 (61)	71 (61)	40 (61)	0.992
Coronary artery disease	22 (12)	14 (12)	8 (12)	0.975
Atrial fibrillation	6 (3)	2 (2)	4 (6)	0.248
Diabetes mellitus	32 (17)	23 (20)	9 (14)	0.303
Hyperlipidemia	23 (13)	15 (13)	8 (12)	0.891
ICH	25 (14)	13 (11)	12 (18)	0.181
TIA/ischemic stroke	28 (15)	15 (13)	13 (20)	0.215
Alcoholism	35 (19)	22 (19)	13 (20)	0.883
Smoking	38 (21)	25 (21)	13 (20)	0.789
Pre‐antihypertensives	67 (37)	43 (37)	24 (36)	0.958
Oral anticoagulants	4 (2)	3 (3)	1 (2)	0.991
Antiplatelets	35 (19)	22 (19)	13 (20)	0.883
Clinical features				
Systolic BP (mmHg)	153.59 ± 23.46	155.37 ± 24.89	150.44 ± 20.50	0.173
Diastolic BP (mmHg)	84.00 (76.00–96.00)	87.00 (77.25–98.75)	80.00 (72.00–93.00)	0.018
NIHSS score at baseline	4 (1–15)	3 (1–12)	8 (2–20)	0.022
GCS score at baseline	14 (11–15)	14 (12–15)	14 (10–15)	0.140
Neurosurgery performed	53 (29)	38 (33)	15 (23)	0.163
Antihypertensive therapy	129 (37)	83 (72)	46 (70)	0.791
ICH findings				
Hematoma volume (cm^3^)	27.02 (14.04–51.86)	26.55 (12.22–51.30)	26.94 (16.76–52.18)	0.374
Location of hematoma				
Frontal lobe	88 (48)	58 (50)	30 (45)	0.592
Parietal lobe	74 (40)	48 (41)	26 (39)	0.829
Temporal lobe	86 (47)	56 (48)	30 (45)	0.754
Occipital lobe	54 (30)	33 (28)	21 (32)	0.607
Insular lobe	1 (1)	1 (1)	0 (0)	1.000
Cerebellum	1 (1)	0 (0)	1 (2)	0.771
Intraventricular extension	51 (28)	30 (26)	21 (32)	0.371

*Note*: Data are mean ± SD, *n* (%), or median (IQR).

Abbreviations: BP, Blood pressure; GCS, Glasgow Coma Scale; ICH, intracerebral hemorrhage; NIHSS, National Institutes of Health Stroke Scale; TIA, transient ischemic attack.

Severe SVD burden was identified in 66 (36%) participants. Patients with severe SVD burden were older (77 vs 68, *p* < 0.001), and had lower median admission diastolic blood pressure (80 vs 87, *p* = 0.018) and higher NIHSS score (8 vs 3, *p* = 0.022). The demographic and clinical characteristics in each group are shown in Table 1.

### 3.2 Regression analysis for total SVD burden

The univariate logistic regression analysis showed that more patients with severe SVD had unfavorable outcomes at 90‐day (59% vs 31%, *p* < 0.001) compared with the patients without severe SVD (Table 2). The multivariate logistic regression analysis was used to adjust for confounding factors. The results showed that SVD burden (adjusted *p* < 0.001; OR, 2.74; 95% CI, 1.61–4.67) was still an independent factor for unfavorable outcomes. No significant difference was found in HE and neurological deterioration in 7 days and mortality at 90‐day between the two groups (*p* > 0.05 each) (Table 3).

**TABLE 2:** CT‐based total SVD burden and clinical outcomes within 90 days

Clinical outcome	SVD burden score		Unadjusted analysis	
	Nonsevere	Severe	OR (95% CI)	*p* value
Hematoma expansion^a^	10 (9)	8 (12)	1.29 (0.81–2.05)	0.289
Neurological deterioration	28 (24)	21 (32)	1.19 (0.87–1.63)	0.274
Unfavorable outcome	36 (31)	39 (59)	2.06 (1.50–2.82)	<0.001
Mortality	13 (11)	9 (14)	1.22 (0.80–1.85)	0.357

*Note*: Data are *n* (%).

^a^ A total of 157 patients were included in the analysis of hematoma expansion.

**TABLE 3:** Multiple logistic regression analysis between individual neuroimaging markers and outcome

	Hematoma expansion^a^	Neurological deterioration	Unfavorable outcome	Mortality
SVD burden score				
Adjusted *p* value	0.273	0.122	<0.001	0.849
Adjusted OR (95% CI)	1.36 (0.79–2.36)	1.41 (0.91–2.17)	2.74 (1.61–4.67)	0.94 (0.52–1.70)
WML^b^				
Adjusted *p* value	0.160	0.110	<0.001	0.540
Adjusted OR (95% CI)	2.40 (0.71–8.19)	2.17 (0.84–5.60)	8.93 (2.63–30.38)	0.64 (0.15–2.70)
Lacunes^c^				
Adjusted *p* value	0.319	0.472	0.925	0.260
Adjusted OR (95% CI)	0.54 (0.16–1.82)	0.71 (0.29–1.79)	0.94 (0.28–3.16)	0.45 (0.11–1.81)
Cortical atrophy^d^				
Adjusted *p* value	0.091	0.031	0.106	0.053
Adjusted OR (95% CI)	2.79 (0.85–9.14)	2.98 (1.10–8.04)	2.65 (0.81–8.65)	3.53 (0.98–12.69)
Central atrophy^e^				
Adjusted *p* value	0.383	0.669	0.052	0.761
Adjusted OR (95% CI)	0.45 (0.08–2.70)	0.76 (0.21–2.72)	5.46 (0.99–30.17)	1.30 (0.24–6.86)
Frontal ratio^f^				
Adjusted *p* value	0.863	0.817	0.601	0.611
Adjusted OR (95% CI)	1.02 (0.84–1.23)	1.01 (0.90–1.15)	1.05 (0.89–1.23)	0.95 (0.78–1.16)
Third ventricle Sylvian fissure distance^g^				
Adjusted *p* value	0.640	0.214	0.009	0.027
Adjusted OR (95% CI)	0.95 (0.76–1.18)	0.91 (0.79–1.06)	0.76 (0.61–0.93)	0.78 (0.63–0.97)

*Note*: All analyses were adjusted for age, sex, diastolic blood pressure, NIHSS score, GCS score, hematoma volume, and intraventricular hemorrhage. Data are *n* (%) or median (IQR).

Abbreviation: WML, White matter lesion.

^a^ A total of 157 patients were included in the analysis of hematoma expansion.

^b^ Adjusted for lacunes, cortical atrophy, and central atrophy.

^**c**^ Adjusted for WML, cortical atrophy, and central atrophy.

^d^ Adjusted for WML, lacunes, and central atrophy.

^e^ Adjusted for WML, lacunes, and cortical atrophy.

^f^ Adjusted for WML, lacunes, and frontal ratio.

^g^ Adjusted for WML, lacunes, and third ventricle Sylvian fissure distance.

### 3.3 Regression analysis for individual neuroimaging markers

We also analyzed different neuroimaging markers to determine their role in the prognosis of CAA‐ICH.

The univariate analysis showed that cortical atrophy (*p* = 0.026) was the factor affecting hematoma enlargement and it (*p* = 0.003) was also the risk factor for neurological deterioration within 7 days of onset. At 90‐day follow‐up, the factors contributing to unfavorable outcome included WML (*p* < 0.001) and four parameters of cerebral atrophy (all *p* for trend <0.05). Finally, cortical atrophy and the third ventricle Sylvian fissure distance were associated with mortality within 3 months (all *p* for trend <0.05) (Table S1).

The multiple logistic regression analysis of different clinical outcomes was performed to adjust the influence of potential confounders. Severe cortical atrophy was related to neurological deterioration (adjusted *p* = 0.031; OR, 2.98; 95% CI, 1.10–8.04). Severe WML (adjusted *p* < 0.001; OR, 8.93; 95% CI, 2.63–30.38) and shorter third ventricle Sylvian fissure distance (adjusted *p* = 0.009, OR, 0.76; 95% CI, 0.61–0.93) were still the independent factors for unfavorable outcomes. For the mortality in 90 days, third ventricle Sylvian fissure distance (adjusted *p* = 0.027; OR, 0.78; 95% CI, 0.63–0.97) showed statistically significant differences (Table 3).

## 4 DISCUSSION

The present study demonstrated that severe SVD burden on baseline CT could lead to unfavorable functional outcomes at 90‐day in patients with CAA‐related ICH. For the individual neuroimaging markers, severe WML and a shorter third ventricle Sylvian fissure distance might contribute to more unfavorable outcomes at 90‐day. Patients with more severe cortical atrophy were likely to experience neurological deterioration within a week, and a shorter third ventricle Sylvian fissure was the independent risk factor for mortality at 90‐day.

This study was novel in exploring the relationship between SVD and the short‐term prognosis of CAA‐related hemorrhage. In line with previously published findings on spontaneous ICH,^5^, ^7^, ^8^, ^9^, ^10^, ^11^ SVD based on CT might indicate the early prognosis of patients with CAA‐related hemorrhage. Also, higher SVD scores were independently correlated with a higher likelihood of achieving an unfavorable outcome at 3‐month in CAA‐related ICH. Several possible explanations account for this. In CAA, a kind of SVD, the deposition of Aβ may impair the architecture and influence the vasomotor function of vessels, potentially leading to stroke or ICH. The total burden of SVD causes diffuse changes in brain microstructure, which impairs cognition, especially in mid‐to‐late life.^16^ The SVD burden in individuals with CAA is often noticeable, as the CAA progresses, the key brain networks vital for rehabilitation, learning, and cognitive reserve are likely to disrupt. Hence, the severity of neuroimaging abnormalities assessed by CT and MRI scans, such as WML, cortical cerebral microinfarcts^17^ and brain atrophy, indicate the frailty of the underlying brain, which influences susceptibility to, and recovery from, ICH.

In this study, we also found that severe WML was related to unfavorable functional outcomes at 90‐day, which was similar to the results of a meta‐analysis.^18^ WML manifests the loss of vascular supply and blood–brain barrier integrity, demyelination, arteriosclerosis, myelin loss, gliosis, venous collagen tissue hyperplasia, and spongiosis.^19^, ^20^ In patients with CAA, WML is related to vascular Aβ load, damaging the integrity of vascular supply and blood–brain barriers and leading to microaneurysms or fibrinoid necrosis. Long‐term ischemia and hypoxia in brain tissue leads to demyelination of white matter, which impacts connectivity and plasticity of the brain.^21^, ^22^, ^23^ Hence, on the one hand, reduced neuroplasticity may have an adverse impact on the neurological function recovery after ICH. On the other hand, WML is linked to the degeneration of deep medullary veins, which might alter hemodynamics, cause interstitial fluid reflux,^24^ and may affect the clearance of hematoma and perihematomal edema. In a nutshell, these pathological manifestations of preexisting leukoencephalopathy may affect hematoma absorption and neurological rehabilitation, thus affecting the prognosis of patients.

After adjusting for known predictors of spontaneous ICH, significant associations of severe cerebral atrophy with END, unfavorable functional outcome, and mortality were still observed, which was in line with most previous data.^25^ In our study, we also found that age had an effect on mortality at 3 months, even after adjusting for imaging markers and other indicators. Firstly, cortical thinning appeared to be an MRI feature of CAA, which also provided evidence of neurodegeneration. Brain atrophy in CAA‐ICH might, in part, represent neurodegeneration processes, which were characterized by the death of neurons and supporting cells in the neurovascular unit, accompanied by progressive deterioration of cognitive and motor function attributable to functional outcome detrimentally.^26^ Secondly, in previous studies, the decrease of deep medullary veins has been linked to cerebral atrophy, which may be related to the neurodegenerative changes occurring in venules over time.^27^ In patients with CAA, vascular dysfunction has also been found to lead to gray matter disease, contributing to cortical atrophy.^28^, ^29^ The impaired vasodilation may further cause cortical and subcortical microcirculation disturbance, thus exacerbating edema formation. However, some previous studies showed different results. Kwon et al.^30^ found that mild or moderate cerebral atrophy exhibited protective effects in patients with moderate‐volume basal ganglia hemorrhage. This discrepancy might be due to the differences in the study population. In this study, the hematoma of CAA‐ICH was in the superficial location such as cortical and subcortical regions, which manifested little mass effects. On the contrary, in patients with larger hematoma volume and deeply located ICH, mild or moderate atrophy might partly offset the mechanical damage due to the mass effect caused by hematoma, edema, increased intracranial pressure, and herniation; also, the compensatory effects would be more obvious in the early phase of ICH.

This study had several limitations. First, the results might be affected by some bias related to the small sample size and data from retrospective studies. Second, some SVD markers, such as enlarged perivascular spaces, cerebral microbleeds, cortical cerebral microinfarcts, cerebral deep medullary veins, and cortical superficial siderosis, were excluded from our analysis restricted to CT scans. However, our results might provide a reference for clinicians to formulate a more comprehensive and individualized treatment in the hyperacute and acute stages of CAA‐ICH or lobar hemorrhage highly suspicious of CAA. Last but not least, it was a retrospective study, and hence cognitive assessment was ignored. However, cognitive decline was common among survivors after CAA‐ICH and closely linked with the combination of CAA and hypertensive microvascular disease.^31^

## 5 CONCLUSIONS

Higher total SVD burden based on CT was independently associated with 90‐day functional outcome in patients with CAA‐ICH. Taking cerebral SVD on baseline CT into account when determining prognosis in patients with acute CAA‐ICH is promising. Further large‐scale prospective studies are warranted to confirm our findings and also to elucidate the pathophysiological mechanism between SVD and CAA‐ICH.

## AUTHOR CONTRIBUTIONS

Mengke Zhang, Ruiwen Che, Xunming Ji designed the study. Hailiang Sun, Jin Ma, Wenbo Hu, Milan Jia, Changhong Ren, Chuanjie Wu, and Xin liu collected the data. Mengke Zhang analyzed the data. Mengke Zhang drafted the manuscript. Ruiwen Che, Wenbo Zhao, and Xunming Ji revised the manuscript.

## FUNDING INFORMATION

This study was funded by the National Natural Science Foundation of China (No. 82001257), the National Natural Science Foundation of China (No. 82027802) and the Natural Science Foundation of China (No. 81971114).

## Supporting information


Table S1.

## Data Availability

All supporting data contributing to this study can be provided from the corresponding author on reasonable request.

## References

[1] Charidimou A , Boulouis G , Gurol ME , et al. Emerging concepts in sporadic cerebral amyloid angiopathy. Brain. 2017;140(7):1829‐1850. 28334869 10.1093/brain/awx047 PMC6059159

[2] Charidimou A , Gang Q , Werring DJ . Sporadic cerebral amyloid angiopathy revisited: recent insights into pathophysiology and clinical spectrum. J Neurol Neurosurg Psychiatry. 2012;83(2):124‐137. 22056963 10.1136/jnnp-2011-301308

[3] Andreas Charidimou TI , Moulin S , Biffi A , et al. Brain hemorrhage recurrence, small vessel disease type, and cerebral microbleeds: a meta‐analysis. Neurology. 2017;89(8):820‐829. 28747441 10.1212/WNL.0000000000004259 PMC5580863

[4] Mehndiratta P , Manjila S , Ostergard T , et al. Cerebral amyloid angiopathy‐associated intracerebral hemorrhage: pathology and management. Neurosurg Focus. 2012;32(4):E7. 10.3171/2012.1.FOCUS11370 22463117

[5] Xu T , Feng Y , Wu W , et al. The predictive values of different small vessel disease scores on clinical outcomes in mild ICH patients. J Atheroscler Thromb. 2021;28(9):997‐1008. 33551444 10.5551/jat.61267 PMC8532058

[6] Xu M , Cheng Y , Song Q , et al. Total burden of cerebral small vessel disease in recurrent ICH versus first‐ever ICH. Aging Dis. 2019;10(3):570‐577. 31165001 10.14336/AD.2018.0804 PMC6538213

[7] Sato S , Delcourt C , Heeley E , et al. Significance of cerebral small‐vessel disease in acute intracerebral hemorrhage. Stroke. 2016;47(3):701‐707. 26846860 10.1161/STROKEAHA.115.012147

[8] Rodrigues MA , Samarasekera NE , Lerpiniere C , et al. Association between computed tomographic biomarkers of cerebral small vessel diseases and long‐term outcome after spontaneous intracerebral hemorrhage. Ann Neurol. 2021;89(2):266‐279. 33145789 10.1002/ana.25949 PMC7894327

[9] Park YS , Chung MS , Choi BS . MRI assessment of cerebral small vessel disease in patients with spontaneous intracerebral hemorrhage. Yonsei Med J. 2019;60(8):774‐781. 31347333 10.3349/ymj.2019.60.8.774 PMC6660438

[10] Lioutas VA , Wu B , Norton C , Helenius J , Modak J , Selim M . Cerebral small vessel disease burden and functional and radiographic outcomes in intracerebral hemorrhage. J Neurol. 2018;265(12):2803‐2814. 30242743 10.1007/s00415-018-9059-5

[11] Hostettler IC , Schwarz G , Ambler G , et al. Cerebral small vessel Disease and functional outcome prediction after intracerebral hemorrhage. Neurology. 2021;96(15):e1954‐e1965. 33627495 10.1212/WNL.0000000000011746

[12] Chen SJ , Tsai HH , Tsai LK , et al. Advances in cerebral amyloid angiopathy imaging. Ther Adv Neurol Disord. 2019;12:1756286419844113. 31105769 10.1177/1756286419844113 PMC6501479

[13] Linn J , Halpin A , Demaerel P , et al. Prevalence of superficial siderosis in patients with cerebral amyloid angiopathy. Neurology. 2010;74(17):1346‐1350. 20421578 10.1212/WNL.0b013e3181dad605 PMC2875936

[14] Greenberg SM , Ziai WC , Cordonnier C , et al. 2022 guideline for the Management of Patients with Spontaneous Intracerebral Hemorrhage: a guideline from the American Heart Association/American Stroke Association. Stroke. 2022;53(7):e282‐e361. 35579034 10.1161/STR.0000000000000407

[15] Anderson CS , Heeley E , Huang Y , et al. Rapid blood‐pressure lowering in patients with acute intracerebral hemorrhage. N Engl J Med. 2013;368(25):2355‐2365. 23713578 10.1056/NEJMoa1214609

[16] Jansen MG , Griffanti L , Mackay CE , et al. Association of cerebral small vessel disease burden with brain structure and cognitive and vascular risk trajectories in mid‐to‐late life. J Cereb Blood Flow Metab. 2022;42(4):600‐612. 34610763 10.1177/0271678X211048411 PMC8943617

[17] Zwartbol MH , Rissanen I , Ghaznawi R , et al. Cortical cerebral microinfarcts on 7T MRI: risk factors, neuroimaging correlates and cognitive functioning ‐ the Medea‐7T study. J Cereb Blood Flow Metab. 2021;41(11):3127‐3138. 34187229 10.1177/0271678X211025447 PMC8543666

[18] Yu Z , Zheng J , Guo R , Ma L , You C , Li H . Prognostic significance of leukoaraiosis in intracerebral hemorrhage: a meta‐analysis. J Neurol Sci. 2019;397:34‐41. 30580053 10.1016/j.jns.2018.12.022

[19] Gouw AA , Seewann A , van der Flier WM , et al. Heterogeneity of small vessel disease: a systematic review of MRI and histopathology correlations. J Neurol Neurosurg Psychiatry. 2011;82(2):126‐135. 20935330 10.1136/jnnp.2009.204685

[20] Hamanaka G , Ohtomo R , Takase H , Lok J , Arai K . White‐matter repair: interaction between oligodendrocytes and the neurovascular unit. Brain Circ. 2018;4(3):118‐123. 30450418 10.4103/bc.bc_15_18 PMC6187946

[21] Wada R , Aviv RI , Fox AJ , et al. CT angiography "spot sign" predicts hematoma expansion in acute intracerebral hemorrhage. Stroke. 2007;38(4):1257‐1262. 17322083 10.1161/01.STR.0000259633.59404.f3

[22] Pantoni L . Cerebral small vessel disease: from pathogenesis and clinical characteristics to therapeutic challenges. Lancet Neurol. 2010;9(7):689‐701. 20610345 10.1016/S1474-4422(10)70104-6

[23] Zhao W , Wu C , Stone C , Ding Y , Ji X . Treatment of intracerebral hemorrhage: current approaches and future directions. J Neurol Sci. 2020;416:117020. 32711191 10.1016/j.jns.2020.117020

[24] Zhang R , Huang P , Jiaerken Y , et al. Venous disruption affects white matter integrity through increased interstitial fluid in cerebral small vessel disease. J Cereb Blood Flow Metab. 2021;41(1):157‐165. 32065078 10.1177/0271678X20904840 PMC7747165

[25] Herweh C , Prager E , Sykora M , Bendszus M . Cerebral atrophy is an independent risk factor for unfavorable outcome after spontaneous supratentorial intracerebral hemorrhage. Stroke. 2013;44(4):968‐971. 23412376 10.1161/STROKEAHA.111.670901

[26] Smith EE . Cerebral amyloid angiopathy as a cause of neurodegeneration. J Neurochem. 2018;144(5):651‐658. 28833176 10.1111/jnc.14157

[27] Ao DH , Zhang DD , Zhai FF , et al. Brain deep medullary veins on 3‐T MRI in a population‐based cohort. J Cereb Blood Flow Metab. 2021;41(3):561‐568. 32312169 10.1177/0271678X20918467 PMC7922755

[28] Fotiadis P , van Rooden S , van der Grond J , et al. Cortical atrophy in patients with cerebral amyloid angiopathy: a case‐control study. Lancet Neurol. 2016;15(8):811‐819. 27180034 10.1016/S1474-4422(16)30030-8 PMC5248657

[29] van Opstal AM , van Rooden S , van Harten T , et al. Cerebrovascular function in presymptomatic and symptomatic individuals with hereditary cerebral amyloid angiopathy: a case‐control study. Lancet Neurol. 2017;16(2):115‐122. 27989553 10.1016/S1474-4422(16)30346-5 PMC5505183

[30] Kwon SM , Choi KS , Yi HJ , et al. Impact of brain atrophy on 90‐day functional outcome after moderate‐volume basal ganglia hemorrhage. Sci Rep. 2018;8(1):4819. 29555930 10.1038/s41598-018-22916-3 PMC5859038

[31] Pasi M , Sugita L , Xiong L , et al. Association of cerebral small vessel disease and cognitive decline after intracerebral hemorrhage. Neurology. 2021;96(2):e182‐e192. 33067403 10.1212/WNL.0000000000011050 PMC7905779

