# Supplementary material for: Neuroimaging biomarkers of small vessel disease in cerebral amyloid angiopathy‐related intracerebral hemorrhage
Source: CNS Neurosci Ther. 2023 Feb 5;29(5):1222–8. doi: 10.1111/cns.14098 (PMC10068469; doi:10.1111/cns.14098)
Supplement: Supplementary file 1 — Table S1. [file CNS-29-1222-s001.docx]

**SUPPLEMENTARY MATERIAL**

**Table S1. CT-based individual SVD neuroimaging markers and clinical outcomes**

|  | **Hematoma expansion**^†^ | ***P* value** | **Neurological deterioration** | ***P* value** | **Unfavorable**  **outcome** | ***P* value** | **Mortality** | ***P* value** |
| --- | --- | --- | --- | --- | --- | --- | --- | --- |
| **WMH 0.180 0.052 ＜0.001 0.616** | | | | | | | | |
| **Nonsevere** | 10（8） |  | 28（22） |  | 35（28） |  | 14（11） |  |
| **Severe** | 8（14） |  | 21（36） |  | 40（69） |  | 8（14） |  |
| **Lacunes 0.393 0.226 0.322 0.169** | | | | | | | | |
| **Nonsevere** | 11（12） |  | 28（31） |  | 34（37） |  | 14（15） |  |
| **Severe** | 7（8） |  | 21（23） |  | 41（45） |  | 8（9） |  |
| **Atrophy: visual evaluation** | | | | | | | | |
| **Cortical 0.026 0.003 ＜0.001 ＜0.001** | | | | | | | | |
| Nonsevere | 10（7） |  | 29（21） |  | 43（31） |  | 9（7） |  |
| Severe | 8（18） |  | 20（44） |  | 32（71） |  | 13（29） |  |
| **Central 0.826 0.438 ＜0.001 0.163** | | | | | | | | |
| Nonsevere | 16（10） |  | 41（26） |  | 56（35） |  | 17（11） |  |
| Severe | 2（8） |  | 8（33） |  | 19（79） |  | 5（21） |  |
| **Atrophy: linear measurements** | | | | | | | | |
| Frontal ratio | 34.21 ± 3.07 | **0.780** | 34.73 ± 3.81 | **0.088** | 35.15（32.39–36.98） | **0.001** | 34.72 ± 3.25 | **0.296** |
| Third ventricle Sylvian fissure distance | 39.19 ± 3.37 | **0.362** | 39.02 ± 3.55 | **0.053** | 38.60（37.00–40.30） | **＜0.001** | 37.58 ± 3.44 | **0.001** |

^†^A total of 157 patients included in the analysis of hematoma expansion.

WML, White matter lesion. Data are *n* (%) or median (IQR).
